# Supplementary material for: Cellulose Nanocrystal Surface Cationization: A New Fungicide with High Activity against Phycomycetes capsici
Source: Molecules. 2019 Jul 4;24(13):2467. doi: 10.3390/molecules24132467 (PMC6651198; doi:10.3390/molecules24132467)
Supplement: Supplementary file 1 [file molecules-24-02467-s001.pdf]

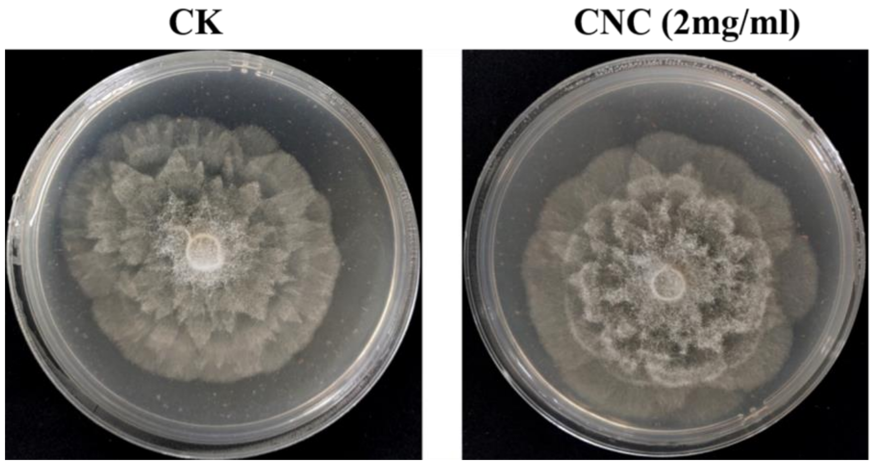

Figure S-1: The antifungal activity of cellulose nanocrystal (CNC) *against Phytophthora capsici*. CK is the water group. CNC is the cellulose nanocrystal.

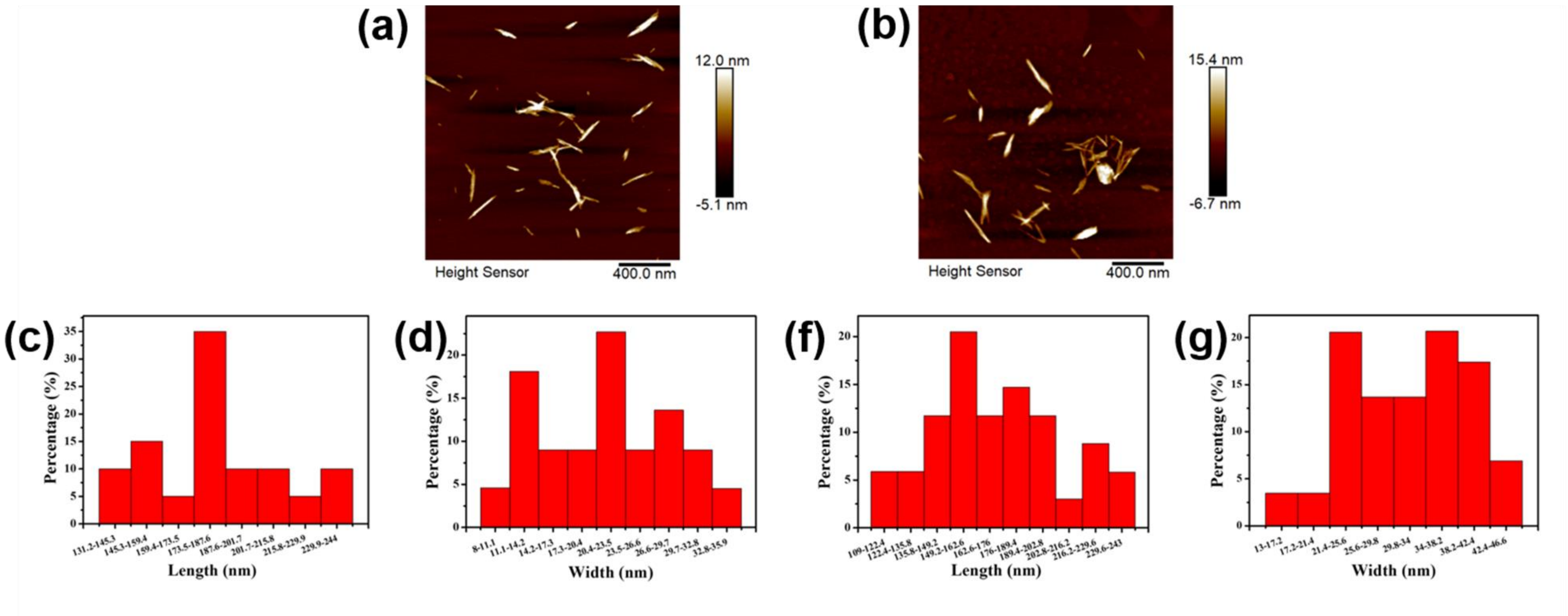

Figure S-2: AFM images of CNC (a) and CNC@CTAB (b). CNC length (c) and width (d) statistics. CNC@CTAB length (e) and width (f) statistics. CNC is the cellulose nanocrystal. CNC@CTAB is a material formed by the CTAB and CNC.
